# Evaluating the effects of antimicrobial drug use on the ecology of antimicrobial resistance and microbial community structure in beef feedlot cattle

**DOI:** 10.3389/fmicb.2022.970358

**Published:** 2022-12-13

**Authors:** Enrique Doster, Lee J. Pinnell, Noelle R. Noyes, Jennifer K. Parker, Cameron A. Anderson, Calvin W. Booker, Sherry J. Hannon, Tim A. McAllister, Sheryl P. Gow, Keith E. Belk, Paul S. Morley

**Affiliations:** ^1^Department of Microbiology, Immunology, & Pathology, Colorado State University, Fort Collins, CO, United States; ^2^Veterinary Education, Research, and Outreach Program, Texas A&M University, Canyon, TX, United States; ^3^Department of Veterinary Population Medicine, University of Minnesota, Minneapolis, MN, United States; ^4^Feedlot Health Management Services, Okotoks, AB, Canada; ^5^Agriculture and Agri-Food Canada, Lethbridge, AB, Canada; ^6^Public Health Agency of Canada, Saskatoon, SK, Canada

**Keywords:** resistome, microbiome, microbial ecology, antimicrobial drug exposure, antimicrobial resistance, cattle, beef feedlot

## Abstract

**Introduction:**

Use of antimicrobial drugs (AMDs) in food producing animals has received increasing scrutiny because of concerns about antimicrobial resistance (AMR) that might affect consumers. Previously, investigations regarding AMR have focused largely on phenotypes of selected pathogens and indicator bacteria, such as *Salmonella enterica* or *Escherichia coli*. However, genes conferring AMR are known to be distributed and shared throughout microbial communities. The objectives of this study were to employ target-enriched metagenomic sequencing and 16S rRNA gene amplicon sequencing to investigate the effects of AMD use, in the context of other management and environmental factors, on the resistome and microbiome in beef feedlot cattle.

**Methods:**

This study leveraged samples collected during a previous longitudinal study of cattle at beef feedlots in Canada. This included fecal samples collected from randomly selected individual cattle, as well as composite-fecal samples from randomly selected pens of cattle. All AMD use was recorded and characterized across different drug classes using animal defined daily dose (ADD) metrics.

**Results:**

Overall, fecal resistome composition was dominated by genes conferring resistance to tetracycline and macrolide-lincosamide-streptogramin (MLS) drug classes. The diversity of bacterial phyla was greater early in the feeding period and decreased over time in the feedlot. This decrease in diversity occurred concurrently as the microbiome represented in different individuals and different pens shifted toward a similar composition dominated by Proteobacteria and Firmicutes. Some antimicrobial drug exposures in individuals and groups were associated with explaining a statistically significant proportion of the variance in the resistome, but the amount of variance explained by these important factors was very small (<0.6% variance each), and smaller than associations with other factors measured in this study such as time and feedlot ID. Time in the feedlot was associated with greater changes in the resistome for both individual animals and composite pen-floor samples, although the proportion of the variance associated with this factor was small (2.4% and 1.2%, respectively).

**Discussion:**

Results of this study are consistent with other investigations showing that, compared to other factors, AMD exposures did not have strong effects on antimicrobial resistance or the fecal microbial ecology of beef cattle.

## Introduction

Antimicrobial resistance (AMR) is one of the most critical global public health issues (World Health Organization (WHO), 2015, 2017, 2019; Centers for Disease Control and Prevention (CDC), 2019, 2020). Misuse and overuse of antimicrobial drugs (AMDs) are commonly attributed as the principal drivers of this problem, and many believe that these practices are accelerating the development and spread of AMR (World Health Organization (WHO), 2015, 2019; Centers for Disease Control and Prevention (CDC), 2019). The use of AMDs in food-producing animals has received increasing criticism and scrutiny regarding this critical issue. Some propose that the use of AMDs within agriculture poses an unacceptable risk to public health because of the potential promotion of AMR, and the potential distribution of resistant bacteria either directly *via* food products, or indirectly through contamination of soil, water, and air (World Health Organization (WHO), 2015, 2019; Centers for Disease Control and Prevention (CDC), 2019).

Traditionally, research and surveillance for AMR have relied on aerobic culture to isolate bacterial isolates which then undergo *in vitro* phenotypic assessments of resistance to various drugs (i.e., using broth microdilution and disk diffusion). While informative, results from these types of studies only provide information about AMR patterns for the very limited number of culturable microorganisms, and for a few AMDs. Culture-based approaches have a limited ability to provide a holistic perspective on resistance in entire microbial communities (microbiomes). Evaluating AMR within an ecological, community-based context is critical, as resistance genes are not limited to a single species or strain but are found within many microbial taxa in the microbiome (e.g., Brinkac et al., 2017; Ghosh et al., 2013). Further, resistance genes can be rapidly transferred between microorganisms *via* horizontal gene transfer or mobile genetic elements (von Wintersdorff et al., 2016). Thus, the entire reservoir of resistance genes (the resistome) can be considered to have a related but potentially separate ecology from the microbiome and should be investigated within the context of whole microbial communities.

The composition of the microbiome and resistome can be investigated in a comprehensive manner without the need to culture specific bacteria through next-generation sequencing (NGS). Previous studies have contributed to characterizing the composition of the resistome and microbiome in beef feedlot environments, however, limited information is available on the influence of commercially-relevant AMD use on the resistome (e.g., Ghosh and LaPara, 2007; Chen et al., 2008; Platt et al., 2008; Kyselková et al., 2015; Noyes et al., 2016b, 2017; Doster et al., 2018; Rovira et al., 2019). The primary goal of this study was to characterize the impact of AMD exposures, in the context of other management and environmental factors, on the fecal microbiome and resistome of beef feedlot cattle. This study used AMR target-enriched metagenomic sequencing and 16S rRNA gene sequencing to investigate the effects of AMD use on the fecal resistome and microbiome of cattle at four Canadian feedlots with detailed AMD use records (Benedict et al., 2013; Noyes et al., 2016a).

## Materials and methods

### Study design and sample collection

Fecal samples were collected during a 3-year longitudinal study of 60,169 cattle housed in 305 randomly selected pens located in Canadian beef feedlot operations, and results regarding *in vitro* susceptibility of fecal *Escherichia coli* and respiratory *Mannheimia haemolytica* isolates have been previously described (Benedict et al., 2015; Noyes et al., 2015, 2016a). Briefly, cattle were enrolled from September 2007 to January 2010 using two-stage random sampling. As cattle arrived at the feedlots, 30% of all newly formed pens were randomly selected for inclusion, and approximately 10% of all cattle housed in selected pens were randomly enrolled. Fecal samples were collected per rectum from individual selected cattle to investigate factors affecting antimicrobial resistance in individuals. Fecal samples also were collected from the floor of pens and used to create composite samples for investigation of (pen) group-level factors that might affect resistance in these group-based samples. Additionally, the ability to use composite samples as an alternative to individually sample cattle was explored.

Individual cattle were sampled twice over the course of the study: during initial processing, which occurred shortly after arrival to the feedlot (first sample), and later in the feeding period (second sample) when cattle were rehandled for various standard management protocols (e.g., for replacement of growth-promoting implants). The timing for obtaining the second samples from cattle varied from 33 to 202 days-on-feed (DOF) with an average of 95.5 DOF (median = 80.0 DOF). Composite fecal samples were collected from floors of pens soon after occupancy (first sample), near the dates when individual animal samples were collected from animals assigned to that pen (second sample time point), and a subset of enrolled were also sampled just prior to slaughter (third sample time point). As previously described (Noyes et al., 2016a), approximately 14 g samples were collected from each of 20 freshly voided fecal pats using a standardized spatial pattern in pens (i.e., walking a Z-shaped pattern from the front corner of the pen to the opposite back corner). These were placed together in a sterile bag and then mixed by hand massage of the bag. After thorough mixing, approximately 10–15 g aliquots were removed, placed in Cary-Blair media and refrigerated for transport to the laboratory (Benedict et al., 2013; Noyes et al., 2016a), placed on ice and then transported to the laboratory for further processing. The intention was that each of the large, 10 g composite samples would represent the larger pen groups. After collection, at the laboratory, fecal samples were processed for aerobic culture and *in vitro* susceptibility testing as previously described, and then archived at −80°C. These samples were archived as described for approximately 10 years prior to being processed for this study. All exposures of the study population to antimicrobial drugs, including parenteral treatments and in-feed exposures, were recorded and standardized across different drug classes using animal defined daily dose estimates (ADD) (Benedict et al., 2015; Noyes et al., 2015, 2016a).

### Sample selection for metagenomic sequencing

For this investigation of the fecal microbiome and resistome, a subset of all individual animal and composited pen-floor fecal samples collected for the previous study were randomly selected for use in this metagenomic sequencing study. The inclusion of both individual animal and composite pen-floor samples allowed for the evaluation of AMR trends both, within individual animals which receive varying amounts of parenteral AMD treatments, and at the pen-level which can be influenced by AMD exposures in >100 cattle housed in each pen. Compositing of pen-floor samples is also much easier to conduct, and it is relevant to understand whether analysis of the resistome in samples obtained from individuals yields similar information to analysis of group-level samples. This provides a comprehensive approach for evaluating the effects of AMD use on the resistome and microbiome in beef feedlot systems. Samples were randomly selected for analysis after stratifying individual cattle based upon cumulative AMD exposures prior to sampling, and stratifying pen-floor samples on the cumulative AMD exposures prior to sampling and also on the timing of collection at the second time point (Supplementary Figure 1). Samples from both the first and second sampling time points had to be available for testing for individual cattle to be eligible for inclusion. Twenty eligible cattle were selected from each of 3 parenteral ADD exposure categories: cattle with no AMD exposure prior to the second time point, those exposed parenterally to 1–4 ADDs prior to the second time point, and those exposed to >4 ADDs parenterally. Thus, the total subset included 60 samples collected from individual cattle sampled at both of the first and second time points. However, of the 120 individual-animal fecal samples selected, only 94 could be included because DNA extraction yield from these archived samples using the large volume extraction protocol (Qiagen PowerMax Soil Kit; Qiagen Laboratories) did not meet the relatively large amounts of DNA required for both target-enriched shotgun sequencing and 16S rRNA amplicon sequencing (target of 9 μg DNA per sample to ensure sufficient quantities were available for multiple attempts at preparation of target enriched shotgun sequencing libraries if needed; the manufacturer’s high input protocol required 3 μg of purified DNA per attempt, Noyes et al., 2017; Agilent, 2021). The need to exclude some samples because of insufficient purified DNA was not associated with important study factors, such as prior antimicrobial exposures, timing of sampling, etc.

For pens to be eligible for inclusion and analysis, archived composite samples had to be available from both the first and second time points. Because the total ADDs of AMD exposures were relatively low among eligible pens, the 6 pens that had the highest total accumulated ADDs were purposively selected for inclusion. The remaining eligible pens were stratified on whether the second sample was collected before or after 100 DOF, and 19 pens were randomly selected from each of these strata for inclusion. As previously described (Noyes et al., 2016a), a limited number of pens were sampled at the third time point, and 10 of these samples were randomly selected for inclusion. Thus, the total subset included 44 samples collected at each of the first and second time points, and 10 samples collected at the third time point (*n* = 98).

### DNA extraction

Genomic DNA was isolated from 5 g of feces using the Qiagen PowerMax Soil Kit (Qiagen Laboratories) according to manufacturer’s instructions, with two exceptions to increase yield. A large sample mass (i.e., 2 × 5 g) was used for DNA extraction with the intent of better potential representation of the resistome and microbiome communities in the fecal samples. First, samples were centrifuged for 5 min in the PowerMax bead tubes, as opposed to the recommended 3 min. Second, samples were eluted using 3 ml as opposed to the recommended 5 ml and were passed through the silica DNA filter twice. To increase DNA concentration, isolated DNA was precipitated with ethanol and 0.3 M sodium acetate, washed with 70% ethanol, and resuspended in 150 μl of PowerMax elution buffer. DNA was then quantified in duplicate using the Qubit 2.0 Fluorometer and dsDNA High Sensitivity Buffer and Reagent kit (Thermo Fischer Scientific) and a final concentration was calculated by averaging the two measurements. DNA was also assayed for quality (A_260_/A_280_ and A_260_/A_230_) using a NanoDrop 1000 Spectrophotometer (Thermo Fischer Scientific). If a sample failed to reach a target yield of 9 μg purified DNA per sample, it was extracted a second time and combined with the first. This large target for extracted DNA was necessary to allow multiple attempts for target-enriched library preparation using the manufacturer’s large input protocol (3 μg per attempt; Agilent, 2021). If there was not 5 g of sample remaining for the second extraction, sterile PBS was used to recover remaining feces off the transport tubes that samples were stored in. The volume of PBS used was dependent upon the weight of the remaining sample; more PBS was used for samples with less weight, in order to reach a total of 5 g (w/v) extraction volume.

### Library preparation and sequencing

The SureSelectXT Reagent Kit for Illumina Paired-End Multiplexed Sequencing Library (Agilent Technologies) was used to prepare samples for target-enriched resistome sequencing (Agilent, 2021). The large input protocol (3ug purified DNA per library; Agilent, 2021) was used with the intent of reducing inter-sample variability that might be associated with very small aliquots, and increasing the likelihood that each library was more representative of the true, average resistome community structure. A custom bait design targeting AMR genes, “MEGaRICH” (Noyes et al., 2017) was used to enrich sequencing libraries for AMR gene sequences. Compared to standard metagenomic sequencing, this bait-capture and enrichment system significantly increases on-target sequencing of previously described AMR genes, as they can often make up <1% of all sequenced DNA in a sample (Noyes et al., 2017). Resulting libraries were pooled in equal proportions based on their molecular weight and DNA concentrations. The pooled library was sequenced at the Denver Genomics and Microarray Core Facility (Denver, CO) on an Illumina NovaSeq 6,000 instrument using paired-end chemistry (2 × 150 bp) and a targeted read depth of 15 million reads per sample.

Extracted DNA (200–500 ng) from each sample (*n* = 218) was sent to the Novogene Corporation for 16S rRNA gene amplification, library preparation, and sequencing. The V4 region of the 16S rRNA gene was amplified using the 515F/806R primer pair [5′-GTGCCAGCMGCCGCGGTAA-3′]/[5′-GGACTACHVGGGTWTCTAAT-3′]. Amplicon sequencing was performed on an Illumina HiSeq 2,500 instrument using paired-end chemistry (2 × 250 bp) and a targeted read depth of 100,000 reads per sample.

### Resistome and microbiome characterization

Target-enriched AMR metagenomic sequencing reads were processed using the AMR++ v2 bioinformatic pipeline and the MEGARes v2 resistance database (Lakin et al., 2017; Doster et al., 2020). A detailed description of MEGARes and the AMR++ pipeline can be found at http://meglab.org. Briefly, reads were trimmed and filtered for quality using trimmomatic (Bolger et al., 2014), and bovine host DNA was identified by aligning trimmed reads to the *Bos Taurus* genome with BWA (Li and Durbin, 2009) and removing those aligned reads. Using BWA, reads were aligned to the MEGARes database and with samtools, alignments were then de-duplicated to remove reads with 100% similarity. For each sample, only genes which had reads aligning to >80% of the reference nucleotide sequence were considered for further analysis. However, reads aligning to genes that require the presence of specific single nucleotide polymorphisms to confer resistance were identified, removed from the downstream statistical analysis, and described separately. Additionally, a list of important AMR gene determinants in human-associated pathogens were identified *a priori* and searched for in all samples. These included genetic determinants classified as Class A carbepenemases [*bla*(IMI), *bla*(SME), *bla*(GES), *bla*(KPC)], Class B carbapenemases [*bla*(NDM), *bla*(IMP), *bla*(cph)], Class D carbapenemases [*bla*(OXA)], extended spectrum betalactamase [*bla*(TEM), *bla*(SHV), *bla*(CTX-M), *bla*(CMY)], streptogramin resistance [*vga*/*vgb*/*vat*], colistin resistance [*mcr*], and multidrug resistance to phenicols, lincosamides, oxazolidinones, pleuromutilins, and streptogramin A antibiotics (PhLOPS_A_) [*cfr*].

16S rRNA amplicon sequence reads were analyzed using QIIME2 v2021.2 (Bolyen et al., 2019). Briefly, all reads were processed for sequence quality and denoised using DADA2 (Callahan et al., 2016), and the resulting amplicon sequence variants (ASVs) were classified using a Bayesian classifier trained on the SILVA v138 database which is implemented in QIIME2 (Quast et al., 2013; Kaehler et al., 2019). Reads that mapped to chloroplast and mitochondrial DNA were then removed from the ASV count table.

### Processing of count matrices

Following read processing with AMR++ or QIIME2, classification count matrices for AMR gene accessions and ASVs, respectively, were imported into R v3.6.1 (R Core Team, 2018). Cumulative sum scaling (CSS; Paulson et al., 2013) was used to normalize feature counts and account for differences in sequencing depth using the R package “metagenomeSeq” (Paulson et al., 2013). Sparsely represented features that were identified in fewer than 5% of samples were removed from further analysis based on published recommendations (Paulson et al., 2013). Resistance data was then summarized to the class and mechanisms level to avoid bias at the “gene” level associated with irregular naming criteria for resistance genes (Hall and Schwarz, 2016). Resistance determinants that affect multiple drug/compound classes (e.g., multi-compound resistance mechanisms such as multidrug efflux pumps) were categorized and analyzed as a separate resistance “class” (multidrug resistance – MDR). Because there is not an efficient method using high-throughput computational processing to confirm the presence of SNPs that are critical for conferring resistance in some specific genes, reads aligning to gene accessions that require SNP confirmation were excluded from statistical analysis. For microbial community analyses, taxonomy was assigned at the level of phylum, class, order, family, genus, and species. In these data, only 54% of ASVs were classified down to the genus level; as such, classification at lower levels are not reported as results at lower taxonomic levels are not considered highly reliable (Peabody et al., 2015), results are only presented at the phylum and order levels. Richness and Shannon’s diversity indices were calculated for all samples at each taxonomic level (e.g., gene, mechanism, phylum, class, etc) using the “vegan” package. Raw classified counts for the microbiome and resistome were analyzed with the “rarecurve” function from the “vegan” package. The resulting rarefaction plots were visually inspected to assess sequencing depth for the microbiome and resistome.

### Statistical analysis

In alignment with the primary study goals, the exposures of interest for statistical analyses that were identified *a priori* were the total ADD exposures for AMDs, and the time that animals were in the feedlot prior to sampling (e.g., arrival vs. second- or third-sampling timepoints). Because the second and third sampling timepoint occurred at a range of DOF timepoints, a separate variable categorizing the DOF at the time of sample collection was also classified into 5 categorical ranges (arrival to 3 DOF, 4–70 DOF, 71–120 DOF, 121–180 DOF, and >180 DOF). The total ADDs of AMD exposures for each animal and pen sample were calculated as the sum of ADDs from all sources prior to sample collection. However, the data regarding exposures to enrolled individuals versus entire pen groups were categorized separately. Total AMD exposures prior to sampling for individual animals were categorized into 3 ordinal levels based on ADDs: low exposure (<10), medium exposure (10–19), and high exposure (>19). Total AMD exposures for pen groups were also categorized into 3 ordinal levels; low exposure (<400), medium exposure (400–1,100), and high exposure (>1,100). Further, separate metadata variables were created to assess the relationship between high parenteral exposure to the two most common drug classes, Tetracycline and macrolides, and significant shifts in the resistome at the second sampling time point. Following the distribution of parenteral tetracycline exposure in individual animal samples, animals were categorized into groups; no exposure (ADD = 0, *n* = 29), and any exposure (ADD >0, *n* = 31). At the pen level, the following ranges were used; no exposure (ADD = 0, *n* = 15), and any exposure to parenteral tetracycline drugs (ADD > 0, *n* = 30). Exposure levels for parenteral macrolide drugs were categorized into just 2 ordinal levels for individual animals: no exposure (ADD = 0, *n* = 29) and any exposure (ADD > 0, *n* = 31). Similarly, pen level exposures could be categorized into samples with no exposure to parenteral macrolide drugs (ADD = 0, *n* = 29), and any parenteral exposure (ADD > 0, *n* = 16). A summary of all categorical metadata calculated from continuous variables and their associated ranges are available in Supplementary material S1.

Diversity indices were statistically compared using the Wilcoxon signed-rank test (“wilcox.test” function in R) for samples from the same animal and “glm” to test differences between sample groups. CSS-normalized counts were Hellinger-transformed (Legendre and Gallagher, 2001) for ordination using the “metaMDS” function from vegan, which employs non-metric multidimensional scaling on Euclidian distances. Analysis of similarities (ANOSIM) (Clarke, 1993) was used to test differences in the composition of microbial communities and resistomes between categorical metadata sample groups (e.g., sample type, total ADD exposure category, parenteral tetracycline exposure category, parenteral macrolide exposure category, arrival vs. second- or third-sampling, and DOF sampling category). For differential abundance testing, metagenomeSeq’s “fitZig” function was used to fit a zero-inflated Gaussian model and compare log2-fold differences (Paulson et al., 2013) in microbiome and resistome features between sampling time, parenteral tetracycline exposure categories, and parenteral macrolide exposure categories. Limma’s “makeContrast” function (Ritchie et al., 2015) were then used for pairwise comparisons. *p*-values were adjusted for multiple tests using the Benjamini-Hochberg procedure (Benjamini and Hochberg, 1995), and alpha = 0.05 was selected as the statistical significance cut-off value. To account for spurious statistically significant differences in low abundance features, only features with an average expression >1% were considered.

To evaluate the correlation between the composition of microbiome and resistome community features, Procrustes analysis was performed using the “protest” function from the vegan package. Microbiome features were analyzed at the level of the ASV, and resistome features were analyzed at the level of ARG Group. Individual and Pen composite samples were analyzed separately, stratifying on sample timing (First, and Second), and AMD exposure category (Low ADDs, Medium ADDs, High ADDs). Distances were calculated as described above and Procrustes analyses was performed NMDS distances to calculate the correlation between the microbiome and resistome in each sample group, with permutational tests for statistical significance.

To further investigate associations between resistome composition and AMD exposures, raw counts were Hellinger-transformed (Legendre and Gallagher, 2001) and redundancy analysis (RDA) was performed on microbial community and resistome composition to further evaluate the potential significance of different AMD use practices using the “rda” function in R. Significance of the correlation between independent variables and the variance in the microbiome and resistome composition were then tested using the “anova” function in R. To characterize the effect of ADD exposure and DOF on the microbiome and resistome, samples were grouped into 17 metadata categories for analysis. Values for ADD exposures were aggregated by route of administration (in-feed vs. parenteral) and by drug class including macrolides-lincosamides-streptogramin (MLS), tetracyclines, phenicol, betalactam, and sulfamethoxazole-trimethoprim combination. Samples were summarized into metadata variables that reflect the amount and type of antimicrobial drug exposure as well as time in the feedlot and days since the most recent parenteral treatment (Supplementary material S2, S3). Variables regarding individual or pen status at the time of sampling included: feedlot ID (1–4), sampling time (first, second, third), DOF at the time of sampling, number of parenteral treatments with AMDs, total ADDs for exposures to all AMDs, ADDs for all parenteral treatments, ADDs for all in-feed exposures, total ADDs for exposures to tetracycline class drugs, total ADDs for tetracycline drugs administered in feed, ADDs for tetracycline drugs administered parenterally, total ADDs for exposures to MLS class drugs, ADDs for MLS drugs administered in feed, ADDs for MLS drugs administered parenterally, ADDs for parenteral exposure to betalactam class drugs, ADDs for parenteral exposures to phenicol class drugs, ADDs for parenteral exposures to sulfonamide class drugs. All variables were included in the starting model for step-wise backward variable selection and ANOVA testing was used to identify a best fitting model.

## Results

### Individual animal sample sequencing metrics

Across the 94 samples, AMR target-enriched metagenomic sequencing yielded an average of 15,926,612 paired-end reads (clusters) per sample (range 3.1–25.2 M per sample, Supplementary material S2). Filtering to improve overall read quality and to exclude bovine host DNA removed an average of 22.9% of reads per sample (range: 3.0%–38.5%). Sequencing of the 16S rRNA gene amplicons across 120 samples resulted in an average of 147,046 reads per sample (range 101,543–208,020 per sample, Supplementary material S3). Following quality filtering, identification of amplicon sequence variants (ASVs) with DADA2, and removal of chloroplast and chimeric sequences, samples averaged 121,970 counts of ASVs (range 83,191–186,619 per sample, Supplementary material S3). Sequencing depth was assessed by plotting rarefaction curves for each individual animal sample and results were indicative of adequate sequencing depth of the resistome (Supplementary Figure 2) and microbiome (Supplementary Figure 3).

### Composite pen-floor sample sequencing metrics

Across the 98 pen-floor composite samples, target-enriched AMR metagenomic sequencing resulted in an average of 16,470,078 paired-end reads (clusters) per sample (range 6.2 M–25.5 M reads per sample, Supplementary material S2). Filtering to improve overall read quality and to exclude bovine host DNA removed an average of 18.7% of reads per sample (range: 3.3–48.0%, Supplementary material S2). 16S rRNA gene sequencing resulted in an average of 197,889 paired-end reads (range 94,433–219,918 per sample, Supplementary material S3). After quality filtering, identification of ASVs with DADA2, samples averaged 153,854 counts of ASVs (range 77,136–181,603 ASVs per sample). Rarefaction plots created from raw count data for the resistome and microbiome of composite pen-floor samples were suggestive of adequate sequencing depth (Supplementary Figures 4, 5).

### Resistome composition

Across the 94 fecal samples collected from individual cattle, an average of 421,355 de-duplicated reads per sample were classified as genetic determinants of AMR (Supplementary material S4). The classifications represented 1,152 different published gene sequences that confer resistance to 18 different drug classes through 60 distinct resistance mechanisms. Across all sampling time points, the 9 most abundant drug classes (or multi-compound mechanisms) were tetracyclines (60.8%), drug and biocide resistance (8.2%), aminoglycosides (7.2%), macrolide-lincosamide-streptrogramin (MLS—5.6%), betalactams (5.5%), sulfonamides (4.2%), phenicols (3.5%), drug and biocide and metal resistance (2.6%), and biocide and metal resistance (1.1%). The remaining nine classes each comprised less than 1% of all normalized counts. Genes conferring resistance to rifampin were identified in 13/94 samples and resistance to fluoroquinolones was identified in only 1/94 fecal samples. Of the genes that confer tetracycline resistance, 89.1% represented tetracycline resistance ribosomal protection proteins, 8.4% were major facilitator superfamily (MFS) efflux pumps, and 2.5% were tetracycline inactivation enzymes. In the second most abundant group of resistance determinants, multi-compound drug and biocide resistance, 39% of alignments drug and biocide MFS efflux pumps and 30.8% represented drug and biocide RND efflux pumps. Across all samples from individual cattle, an average of 50,365 de-duplicated reads per sample were aligned to gene accessions requiring specific SNPs to confer AMR (Supplementary material S5). These counts were excluded from all downstream analyses.

**Figure 1:**
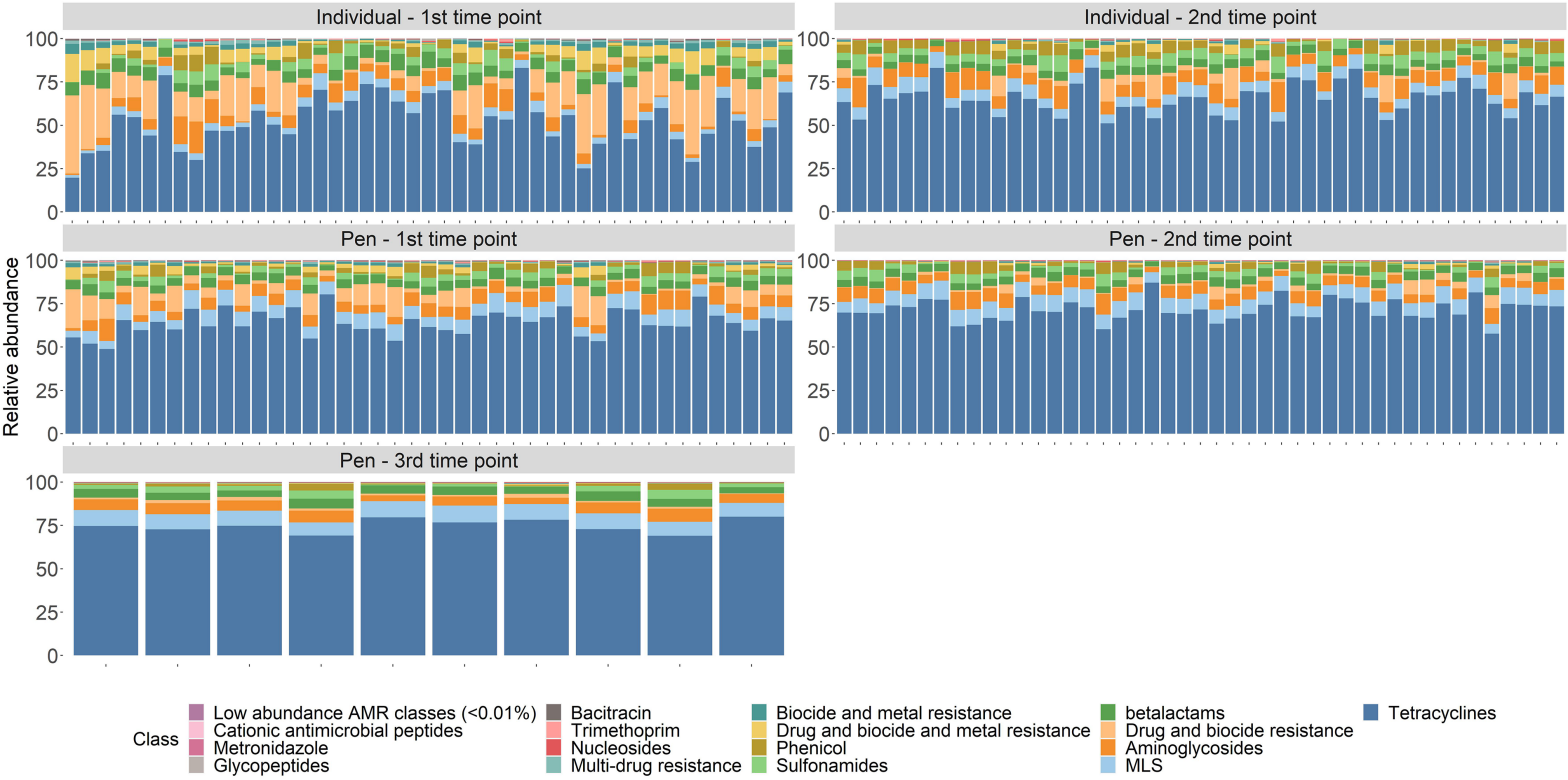
Resistome composition at the drug class level for all samples by sampling time point.

In the 98 composite fecal samples collected from pen-floors (first, second, and 10 samples with a third sampling point), an average of 559,961 de-duplicated reads per sample were classified as genetic determinants of AMR (Supplementary material S4), representing 1,361 genes that confer resistance to 20 different drug classes through 69 distinct resistance mechanisms. Across all sampling time points, the 8 most abundant drug classes or MDR mechanisms were represented by tetracyclines (69.4%), MLS (8.2%), aminoglycosides (6.2%), betalactams (4.6%), multi-compound drug and biocide resistance (4%), sulfonamides (2.8%), phenicol (2.2%), and drug and biocide and metal resistance mechanisms (1.1%). The remaining 12 classes each consisted of <1% of normalized counts (Figure 1). Gene conferring resistance to rifampin were identified in 15/98 samples, resistance to cationic antimicrobial peptides was identified in 5/98 samples, and fosfomycin, mupirocin, and fluoroquinolone resistance were only identified in a single sample each. Of the genes conferring tetracycline resistance, 89% encoded tetracycline resistance ribosomal protection proteins and 8.7% encoded for MFS efflux pumps with the remaining 2.4% associated with tetracycline inactivation enzymes. In the second most abundant resistance class, MLS, the two most abundant resistance mechanisms were 23S rRNA methyltransferases (49.7%) and MLS resistance MFS efflux pumps (24.3%). In composite fecal samples, an average of 52,585 de-duplicated reads per sample were aligned to gene accessions requiring specific SNPs to confer AMR and were excluded from further analyses (Supplementary material S5).

### Changes in resistome composition over time

At the first sampling time point, ANOSIM testing revealed a significant difference between the resistome composition of individual animals compared to pen floor composite samples (class level: ANOSIM R = 0.14, *p* = 0.001; mechanism level: ANOSIM R = 0.16, *p* = 0.001). While a difference in resistome composition between sample types was still significant at the second sampling time point, the dissimilarity indices were lower (class level: ANOSIM R = 0.13, *p* = 0.001; mechanism level: ANOSIM R = 0.12, *p* = 0.001). Due to significant differences in sample type, results are reported separately for individual and pen floor samples.

**Figure 2:**
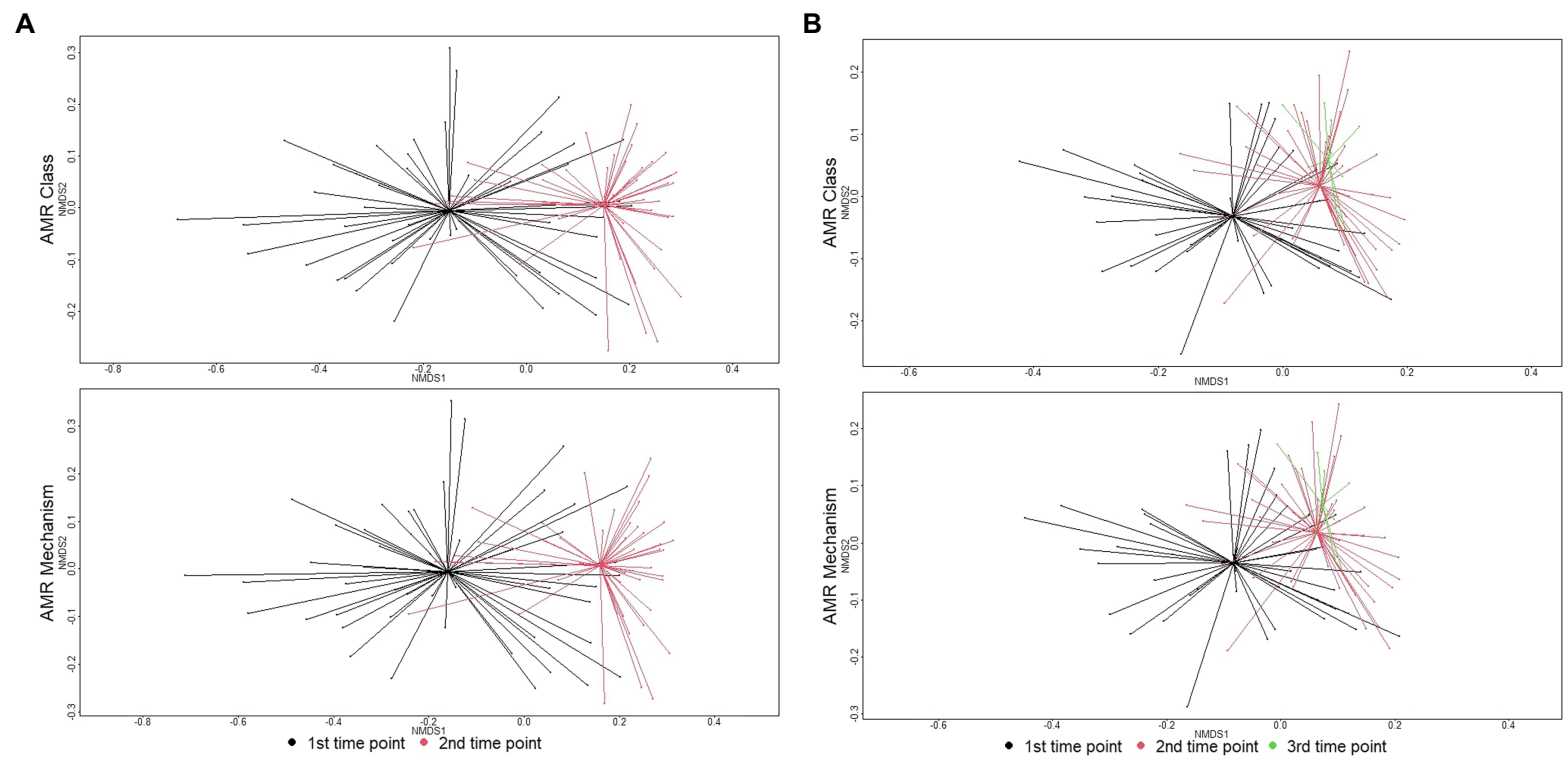
Ordination comparing resistome composition at the AMR drug class and resistance mechanism, using non-metric multidimensional scaling (NMDS), for the two study groups at arrival and re-handling samples. **(A)** Separation of resistomes in individual animals between sampling time was statistically significant at the class (ANOSIM R = 0.33, *p* = 0.001) and mechanism levels (ANOSIM R = 0.34, *p* = 0.001). **(B)** Separation of resistomes in pen-floor samples between the first and second sampling times was statistically significant at the class (ANOSIM R = 0.18, *p* = 0.001) and mechanism levels (ANOSIM R = 0.18, *p* = 0.001).

There were significant shifts in richness and Shannon’s diversity indices between sampling time points. At the class level, richness significantly decreased over time in individual animal samples (W = 1507.5, *p* = 0.002) and in pen-floor composite samples (W = 1412.5, *p* < 0.001). Shannon’s diversity was also significantly reduced between sampling points in the individual animal samples (W = 1,654, *p* < 0.001) and in pen floor composite samples at the class level (W = 1,637, *p* < 0.001). Likewise, there were statistically significant shifts in resistome composition over time for fecal samples collected from both individual animals and pen-floor composite samples. These temporal shifts in resistome composition were greater among samples collected from individual animals (class level: ANOSIM R = 0.33, *p* = 0.001; mechanism level: ANOSIM R = 0.34, *p* = 0.001; Figure 2A) than for pen-floor composite samples (class level: ANOSIM R = 0.18, *p* = 0.001; mechanism level: ANOSIM R = 0.18, *p* = 0.001; Figure 2B). Temporally associated differences in the relative abundance of drug classes were more prominent among individual animal samples than pen-floor composites, particularly due to decreases in the second most abundant resistance class, multi-compound drug and biocide antimicrobial resistance genes (ARGs). The pen-level resistome was dominated by tetracycline, and MLS resistance at the first sampling time point, and by the second sampling time point tetracycline resistance made up a greater proportion of the resistome in both sample types and significant shifts were limited to the drug classes in lower abundance (Figure 1). Of the 8 drug classes comprising greater than 1% of the resistome, 8 were differentially abundant in individual animal samples while only 2 were differentially abundant in pen-floor composite samples (Supplementary material S6). Interestingly, shifts in the composition of individual animal resistomes were primarily the result of significant increases in the relative abundance of the three most prevalent drug classes (tetracyclines, MLS, and sulfonamides). In contrast, the relative abundance of these three drug classes did not change over time in pen-floor composite samples (Supplementary material S7). Instead, the 2 drug classes with significant changes in pen-floor composite samples were all associated with less prevalent drug classes that decreased in relative abundance. Notably classes consisting of multi-drug resistance, drug and biocide resistance, and drug and biocide and metal resistance mechanisms all decreased significantly over time in both individual animal samples and pen-floor composite samples.

### AMD exposures in the study population

AMD exposures for the entire study population have previously been described for the entire study population (Benedict et al., 2015; Noyes et al., 2015). For the subset of individuals and pens that were included in this study, average AMD exposures at the second timepoint in individual cattle selected for this study was 12.7 total ADDs (range 5.9–24.6 ADD), which included an average parenteral exposure of 3.1 ADDs (range 0–7 ADD—Table 1). Parenteral exposures were also dominated by tetracycline (1.6 ADD) and macrolide drugs (1.2 ADD), followed by phenicol (0.2 ADD), and sulfonamide drugs (0.1 ADD) on average. The majority of in-feed AMD exposures were to tetracyclines (average 9.6 ADDs per animal), with much lower exposures to MLS compounds (0.02 ADDs). Correspondingly, groups of cattle housed in enrolled pens were exposed to on average of 2,113.8 ADDs per pen (range 9.9–10,113.3) at the second sampling time, consisting mostly of in-feed AMD exposures. In contrast, pens of cattle were exposed to average of 368.3 ADD by parenteral exposures (range 0–1,367 ADD; Table 1). At the pen level, parenteral exposure to tetracycline drugs was most common, with an average of 140.9 ADD per pen (range 0–902). At one of the participating feedlots, parenteral exposure to fluoroquinolone drugs was more common in the enrolled pens (*n* = 6), accumulating an average of 1219.5 ADDs at the second time point. This is in comparison to an average of 57.8 fluoroquinolone ADDs in the other 49 pens. Without the influence of these six pens, tetracyclines made up the largest percentage of parenteral AMD exposures (65%), followed by fluoroquinolone drugs (30%), and betalactam drugs (2%) with MLS, phenicol, and sulfonamide drugs each making up less than 1% of drug exposures.

**Table 1:** Summary statistics for ADD exposure variables, by sample type.

Sample type	Variable	Mean	Median	Min	Max	SD
Individual—1st time point	Total_ADD	0.0	0.0	0.0	0.0	0.0
Individual—1st time point	Total_feed_ADD	0.0	0.0	0.0	0.0	0.0
Individual—1st time point	Total_parenteral_ADD	0.0	0.0	0.0	0.0	0.0
Individual—1st time point	total_tetracycline_ADD	0.0	0.0	0.0	0.0	0.0
Individual—1st time point	total_MLS_ADD	0.0	0.0	0.0	0.0	0.0
Individual—1st time point	feed_MLS_ADD	0.0	0.0	0.0	0.0	0.0
Individual—1st time point	feed_tetracycline_ADD	0.0	0.0	0.0	0.0	0.0
Individual—1st time point	parenteral_tetracycline_ADD	0.0	0.0	0.0	0.0	0.0
Individual—1st time point	parenteral_MLS_ADD	0.0	0.0	0.0	0.0	0.0
Individual—1st time point	parenteral_phenicol_ADD	0.0	0.0	0.0	0.0	0.0
Individual—1st time point	parenteral_sulfonamide_ADD	0.0	0.0	0.0	0.0	0.0
Individual—1st time point	parenteral_betalactams_ADD	0.0	0.0	0.0	0.0	0.0
Individual—1st time point	parenteral_fluoroquinolones_ADD	0.0	0.0	0.0	0.0	0.0
Individual—2nd time point	Total_ADD	12.7	10.5	5.9	24.6	5.6
Individual—2nd time point	Total_feed_ADD	9.6	7.0	3.9	18.7	5.4
Individual—2nd time point	Total_parenteral_ADD	3.1	3.0	0.0	7.0	1.4
Individual—2nd time point	total_tetracycline_ADD	11.2	9.9	5.9	18.7	4.3
Individual—2nd time point	total_MLS_ADD	1.2	0.1	0.0	3.1	1.5
Individual—2nd time point	feed_MLS_ADD	0.0	0.0	0.0	0.1	0.0
Individual—2nd time point	feed_tetracycline_ADD	9.6	6.9	3.9	18.7	5.4
Individual—2nd time point	parenteral_tetracycline_ADD	1.6	2.0	0.0	4.0	1.4
Individual—2nd time point	parenteral_MLS_ADD	1.2	0.0	0.0	3.0	1.5
Individual—2nd time point	parenteral_phenicol_ADD	0.2	0.0	0.0	3.0	0.7
Individual—2nd time point	parenteral_sulfonamide_ADD	0.1	0.0	0.0	3.0	0.4
Individual—2nd time point	parenteral_betalactams_ADD	0.0	0.0	0.0	0.0	0.0
Individual—2nd time point	parenteral_fluoroquinolones_ADD	0.0	0.0	0.0	0.0	0.0
Pen—1st time point	Total_ADD	903.6	118.2	0.0	9469.5	2030.6
Pen—1st time point	Total_feed_ADD	591.2	38.1	0.0	8212.5	1710.9
Pen—1st time point	Total_parenteral_ADD	312.4	21.0	0.0	1326.0	419.9
Pen—1st time point	total_tetracycline_ADD	702.1	64.9	0.0	8230.5	1700.4
Pen—1st time point	total_MLS_ADD	1.2	0.0	0.0	18.0	3.7
Pen—1st time point	feed_MLS_ADD	0.7	0.0	0.0	12.0	2.4
Pen—1st time point	feed_tetracycline_ADD	590.5	38.1	0.0	8212.5	1711.1
Pen—1st time point	parenteral_tetracycline_ADD	111.7	0.0	0.0	1048.0	231.0
Pen—1st time point	parenteral_MLS_ADD	0.5	0.0	0.0	12.0	2.1
Pen—1st time point	parenteral_phenicol_ADD	0.4	0.0	0.0	16.0	2.5
Pen—1st time point	parenteral_sulfonamide_ADD	1.4	0.0	0.0	27.0	5.5
Pen—1st time point	parenteral_betalactams_ADD	0.9	0.0	0.0	24.0	4.3
Pen—1st time point	parenteral_fluoroquinolones_ADD	197.5	0.0	0.0	1218.0	396.5
Pen—2nd time point	Total_ADD	2113.8	1054.6	9.9	10113.3	2959.3
Pen—2nd time point	Total_feed_ADD	1745.5	763.3	9.9	8822.3	2560.5
Pen—2nd time point	Total_parenteral_ADD	368.3	143.0	0.0	1367.0	451.7
Pen—2nd time point	total_tetracycline_ADD	1880.6	1012.5	9.9	8858.3	2546.9
Pen—2nd time point	total_MLS_ADD	8.4	6.3	0.0	33.8	8.8
Pen—2nd time point	feed_MLS_ADD	5.7	0.0	0.0	33.4	7.6
Pen—2nd time point	feed_tetracycline_ADD	1739.8	751.9	9.9	8822.3	2562.5
Pen—2nd time point	parenteral_tetracycline_ADD	140.9	18.0	0.0	902.0	231.4
Pen—2nd time point	parenteral_MLS_ADD	2.7	0.0	0.0	12.0	4.1
Pen—2nd time point	parenteral_phenicol_ADD	2.8	0.0	0.0	27.0	6.4
Pen—2nd time point	parenteral_sulfonamide_ADD	5.0	3.0	0.0	30.0	7.6
Pen—2nd time point	parenteral_betalactams_ADD	7.3	3.0	0.0	46.0	10.3
Pen—2nd time point	parenteral_fluoroquinolones_ADD	209.6	9.0	0.0	1251.0	418.5
Pen—3rd time point	Total_ADD	1340.0	1349.6	349.5	2520.4	741.7
Pen—3rd time point	Total_feed_ADD	1053.0	945.1	349.5	1850.4	507.6
Pen—3rd time point	Total_parenteral_ADD	287.0	380.8	0.0	670.0	260.0
Pen—3rd time point	total_tetracycline_ADD	1228.9	1245.2	339.7	2348.5	631.5
Pen—3rd time point	total_MLS_ADD	27.7	20.8	0.0	112.6	32.4
Pen—3rd time point	feed_MLS_ADD	25.6	15.9	0.0	112.6	32.7
Pen—3rd time point	feed_tetracycline_ADD	1027.5	935.1	339.7	1833.5	508.4
Pen—3rd time point	parenteral_tetracycline_ADD	201.4	203.0	0.0	515.0	207.3
Pen—3rd time point	parenteral_MLS_ADD	2.1	0.0	0.0	9.0	3.5
Pen—3rd time point	parenteral_phenicol_ADD	2.1	0.0	0.0	18.0	5.7
Pen—3rd time point	parenteral_sulfonamide_ADD	3.1	1.5	0.0	14.0	4.5
Pen—3rd time point	parenteral_betalactams_ADD	6.5	4.0	0.0	28.0	8.7
Pen—3rd time point	parenteral_fluoroquinolones_ADD	71.8	6.3	0.0	582.0	180.3

### Potential associations between resistome composition and AMD exposures

Redundancy analysis included investigation of an explanatory variables regarding feedlot identification, 2 variables regarding timing of sampling, and 14 variables characterizing various types of AMD exposures prior to sampling. When including data from all time points for individual animal samples in one model, sampling time point was the only significant variable (*p* < 0.05), but it was only associated with explaining 2.4% of the constrained variance. For the model investigating data from all time points for pen-floor composite samples, sampling time along with three variables describing parenteral exposure to phenicols, macrolides, and sulfonamides were included in the model resulting from step-wise model selection. In all, the sampling time, ADDs for tetracycline exposure, and total ADD exposure were included in the model and were statistically significant (*p* < 0.05), but only accounted for 0.6%, 0.2%, and 0.1% of the constrained variance, respectively. In both of these models, however, unconstrained variance estimates were much greater than constrained variance estimates, suggesting that these results should be interpreted with caution as only a small amount of the variation in the response (resistome) matrix was represented in the model (Legendre and Gallagher, 2001).

Because of the significant shift observed in resistome composition over time, samples collected at the second time point were analyzed separately with RDA. For samples collected from individual animals, the variable representing the days-on-feed (DOF) at the time of sampling was the only statistically significant (*p* < 0.05) variable included in the final model, describing 0.2% of the constrained variance. For pen-floor composite samples, the final RDA model included only two statistically significant variables, parenteral MLS ADD and feedlot ID, explaining only 0.4% and 0.2% of the constrained variance, respectively. Again, the unconstrained variance for this model was much greater than the constrained variance.

The effect of parenteral exposures to either tetracycline or macrolide drugs on the resistome was assessed by comparing samples at the second time point with no parenteral drug exposure to those that were exposed. In samples from individual animals, there was no significant differences in resistome composition between those with or without exposure to parenteral tetracycline drugs. In pen-floor composite samples, however, there was a significant difference between samples based on exposure to parenteral tetracycline (class level: ANOSIM R = 0.13, *p* = 0.03; mechanism level: ANOSIM R = 0.11, *p* = 0.04). To evaluate if these differences in resistome composition were associated with changes in particular features, a ZIG model was created to include the metadata variables; parenteral macrolide exposure and feedlot ID. Results did not reveal significant differential abundances at either the class or mechanism levels. Alternatively, exposure to parenteral macrolide drugs was not associated with significant differences in resistome composition either in individual samples or pen-floor samples.

### Highly important AMR genes

Of the 17 genes identified *a priori* as being important to medicine when expressed in human pathogens, 11 were identified in at least one sample (Supplementary Figure 6); *bla*(IMI), *bla*(KPC), *bla*(SHV), *bla*(CPH), *bla*(NDM), and *mcr* genes were not identified in any samples. Alignments to these medically important genes accounted for 0.4% (415 K / 93.7 M) of all determinants of AMR across all samples (Supplementary material S8). Determinants for betalactamases were the most abundant type of medically important AMR determinant, representing 47% (195 K/415 K) of alignments to these genes. Among these, *bla*(CTX), *bla*(OXA), and *bla*(TEM) were the most abundant, representing 30% (126 K/415 K), 9% (37 K/415 K), and 7% (31 K/415 K) of alignments to medically important AMR determinants, respectively. The alignments to *bla*(CTX) and *bla*(OXA) genes were fairly evenly distributed across most pen floor composite samples (98/98 and 78/98, respectively), but were more clustered in individual animal samples. This clustering of alignments was even stronger for *bla*(TEM) among a smaller number samples (7/94 and 13/98 for individual and pen-floor samples, respectively). Interestingly, 90% of *bla*(OXA) alignments (33 K/37 K) were to OXA-347 (MEGARes gene accession MEG_4750).^1^ There was also an interesting general trend wherein larger numbers of determinants for these 3 gene groups did not cluster in the same samples. That is, samples that had larger number of alignments for one these genes [*bla*(CTX), *bla*(OXA), or *bla*(TEM)] did not have larger numbers of alignments for the other two. Enzymes encoded by these gene determinants are important in members of the ESBL group. *Bla*(OXA) genes have become medically important because they encode for Class D betalactamase enzymes that are active against cephalosporins and carbapenems (Tooke et al., 2019). While these have been commonly identified in *Acinetobacter* species, *bla*(OXA) genes can be found in a variety of bacteria. All alignments to the *bla*(CTX) group were to one of three MEGAREs gene accessions (MEG_2378, MEG_2430, or MEG_2435), which are variants of the CTX-M-9 subgroup. These ESBL belong to Ambler class A beta-lactamases which have become a medical concern in Enterobacteriaceae isolates (Bonnet, 2004).

The *vgb*A (streptogramin B esterase), *vat* (streptogramin A O-acetyltransferase), and *vga* (multidrug ABC efflux pump) genes confer resistance to quinupristin-dalfopristin (Soltani et al., 2000; Jung et al., 2010). This streptogramin class drug combination is especially important for treatment of infections with resistant Gram-positive bacteria, such as methicillin-resistant *Staphylococcus aureus* (MRSA) and vancomycin-resistant *Enterococcus* spp. (VRE). Alignments to this group of genes were the second most abundant among those identified among the set of medically important AMR genes investigated *a priori*. Collectively, they represented 37% (153 K/415 K) of alignments to the subset of medically important genes and were identified in 82% (158/192) of all samples. Alignments to *vgb*A were the most common among the streptogramin class AMR genes, and the second most abundant among the subset of medically important AMR genes (22% of medically important AMR genes, 92 K/415 K). The identification of *vgb*A, *vat* and *vga* were co-located in 24% (23/94) individual animal samples, and 59% (58/98) of pen-floor composite samples.

Other medically important genes were more sparsely identified in the sample set [*cfr*, bla(SME), bla(CMY), bla(IMP), and bla(GES)]. Reads aligning to *cfr* were distributed among the sample set, especially those for *cfr*A, whereas reads aligning to the other ESBL genes listed [bla(SME), bla(CMY), bla(IMP), and bla(GES)] were clustered within a few samples (Supplementary material S8).

### Correlation between composition of the resistome and microbiome community features

Overall, results of Procrustes analyses showed only moderate correlation between the microbiome composition (ASV level) and resistome composition (ARG Group level). For individual animal samples, animals with Low and Medium AMD exposures had significant correlation between microbiome and resistome features (m^2^ = 0.73, *p ≤* 0.01, and m^2^ = 0.70, *p ≤* 0.005, respectively), whereas animals with high AMD exposures did not have significant correlation (m^2^ = 0.67, *p ≤* 0.36). In contrast, for pen-floor composite samples, groups with Low AMD exposures did not have significant correlation between microbiome and resistome communities (m^2^ = 0.50, *p ≤* 0.23), whereas correlations were significant for pen groups with Medium and High AMD exposures (m^2^ = 0.37, *p ≤* 0.001, and m^2^ = 0.77, *p ≤* 0.02, respectively).

### Microbiome composition

**Figure 3:**
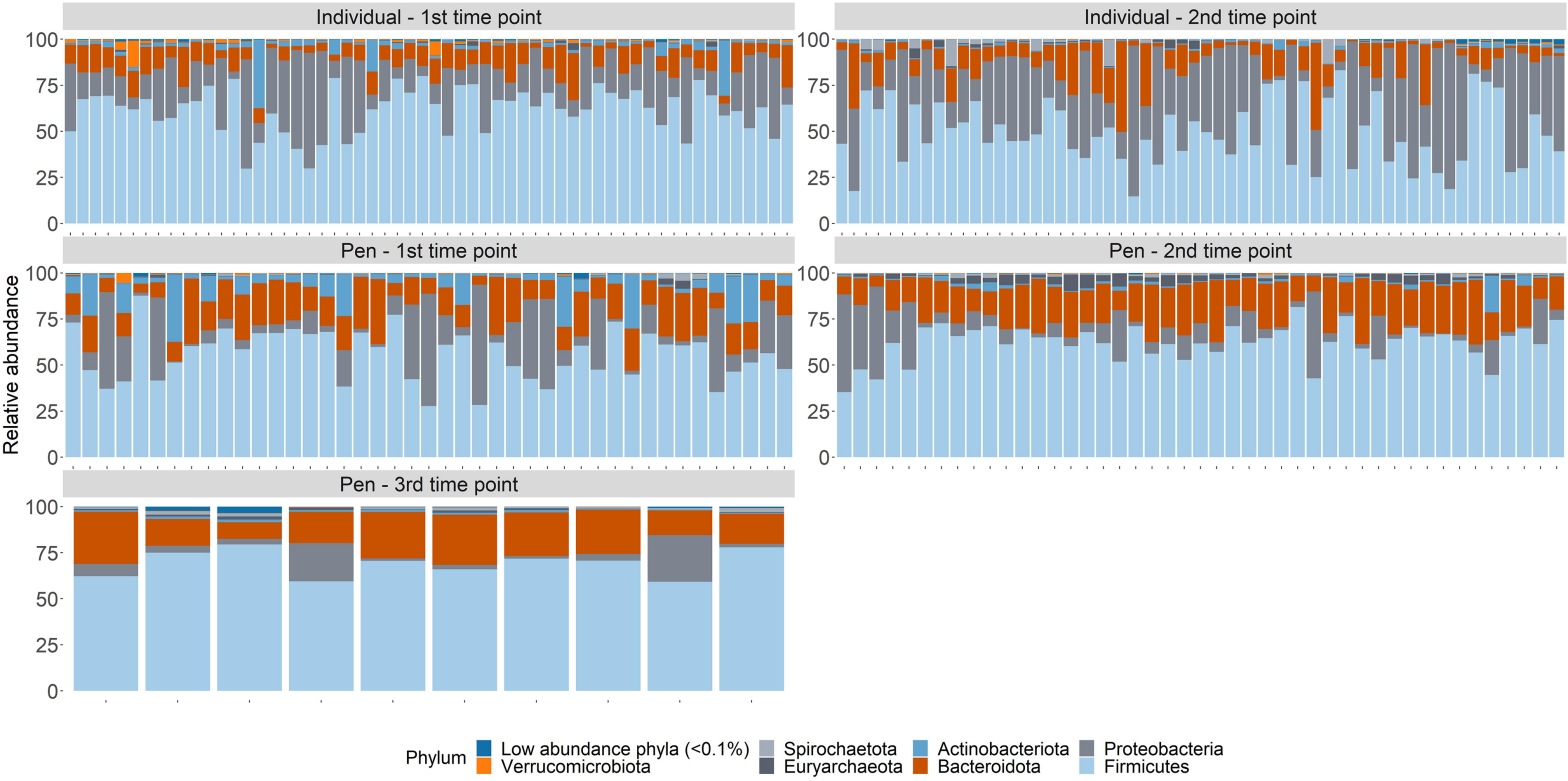
Microbiome composition at the phylum taxonomic level for all samples by sampling time point; arrival at feedlot, a second time point closer to exit of the feeding period, or just prior to shipment to the abattoir.

Across samples from individual animals, taxa from 38 phyla, and 184 orders were represented (Supplementary material S9). Three phyla (Firmicutes, Proteobacteria, and Bacteroidota) accounted for over 96% of all normalized counts (49.9%, 36.3%, and 9.84%, respectively; Figure 3). At the level of order, Pseudomonadales (34.6%), Oscillospirales (15.6%), Lactobacillales (13.5%), Bacteroidales (7.33%), Peptostreptococcales-Tissierellales (5.04%), Lachnospirales (3.99%), Erysipelotrichales (2.51%), Flavobacteriales (2.33%), RF39 (2.11%), Christensenellales (1.7%), Clostridiales (1.59%), Enterobacterales (1.35%), Clostridia_UCG-014 (1.24%), and Bacillales (1.01%) were the most abundant and combined to represent nearly 94% of the microbial community. The remaining 170 orders each made up less than 1% of the overall community.

In pen-floor composite samples, taxa from 37 phyla, and 179 orders were represented. Like individual animal samples, Firmicutes (49.9%), Bacteroidota (18.21%), and Proteobacteria (15.7%) were the three most abundant phyla, albeit in a different order of relative abundance. Additionally, Actinobacteria and Euryachaeota were more abundant within pen-floor composite samples and accounted for 4.76 and 1.43% of the microbial community, respectively (Figure 3). At the order level, Oscillospirales (20.9%), Bacteroidales (17.8%), Pseudomonadales (15.3%), Lactobacillales (13.6%), Lachnospirales(4.78%), Peptostreptococcales-Tissierellales (4.7%), Bifidobacteriales (3.78%), Erysipelotrichales (3.48%), and RF39 (3.35%), and Clostridiales (2.41%) were the most abundant and comprised over 90% of the microbial community. The remaining 169 orders each represented less than 2% of the overall community.

**Figure 4:**
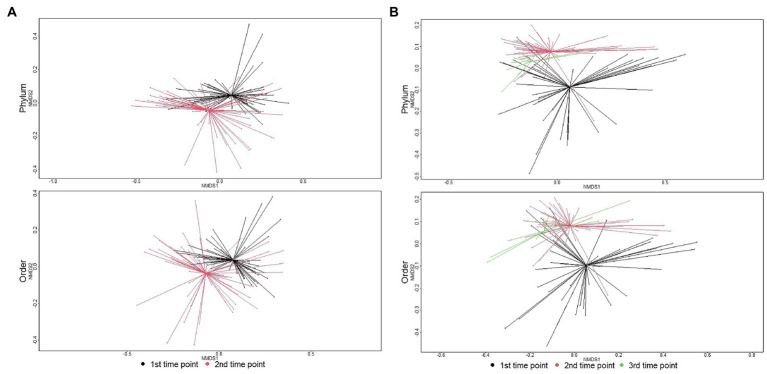
Ordination comparing microbiome composition at the phylum, class, and order levels using non-metric multidimensional scaling (NMDS), for samples collected at arrival, re-handling, or pen-floor samples collected just prior to shipment. **(A)** In individual animals, the separation of resistomes between sampling time was statistically significant at the phylum (ANOSIM R = 0.19, *p* < 0.01), class (ANOSIM R = 0.21, *p* < 0.01), and order (ANOSIM R = 0.22, p < 0.01) taxonomic levels. **(B)** In pen-floor samples, resistome composition shifted significantly between the first and second sampling time and was statistically significant at the phylum (ANOSIM R = 0.08, *p* = 0.01), class (ANOSIM R = 0.12, *p* < 0.01), and order (ANOSIM R = 0.14, *p* < 0.01) taxonomic levels.

### Changes in microbiome composition over time

The microbial community structure of individual animal samples compared to pen composite samples was significantly different at the first sampling point (phylum level: ANOSIM R = 0.22, *p* = 0.001; order level: ANOSIM R = 0.32, *p* = 0.001). This difference in microbiome composition between sample types increased over time and was significantly different at the second sampling time with increased dissimilarity indices (phylum level: ANOSIM R = 0.29, *p* = 0.001; order level: ANOSIM R = 0.31, *p* = 0.001). Due to significant differences by sample type, microbiome results are reported separately for individual and pen floor samples.

The major shift in microbiome composition over time in the feedlot was also evident with richness decreasing between arrival and the second sampling time point for both individual animal and pen-floor samples (*p* < 0.05). Shannon’s diversity, however, only decreased significantly for individual animals at the order level (*p* < 0.05).As demonstrated in the resistome, ANOSIM confirmed that microbial community composition shifted significantly between the first and second time point. Likewise, individual animals had greater shifts in community composition (phylum: ANOSIM R = 0.19, *p* < 0.01; order: ANOSIM R = 0.22, *p* < 0.01) then pen level communities (phylum: ANOSIM R = 0.08, *p* = 0.01; *p* < 0.01; order: ANOSIM R = 0.14, *p* < 0.01; Figure 4).

Of the 4 phyla in individual animal samples with an average relative abundance over 1%, all had significant changes in their relative abundance between sampling time points (*p*-value < 0.05). Proteobacteria, Firmicutes, and Bacteroidota significantly increased in relative abundance between the first and second time point, while Actinobacteria decreased in relative abundance over time(*p* < 0.05) (Supplementary material S10). This pattern was similarly observed in pen-floor composite samples, although with only 2 of the 4 phyla with an average relative abundance over 1%. The relative abundance of Bacteroidota significantly increased over time while Actinobacteriota decreased (Supplementary material S11).

### Potential associations between microbiome composition and AMD exposures

Parallel to the RDA of variance of the resistome composition, RDA of the microbiome composition investigated the effects of 17 explanatory variables, including 14 variables characterizing AMD exposures. Analysis of individual animal samples from both time points identified sampling time, feedlot ID, in-feed MLS ADD, and in-feed Tetracycline as statistically significant (*p* < 0.05). However, the inclusion of these variables in a model only explained 0.8%, 0.6%, and 0.2% of the constrained variance at the phylum level, respectively. For the RDA of pen-floor composite samples from all 3 time points, DOF, total parenteral ADD exposures, and parenteral sulfonamide ADD were statistically significant (*p* < 0.05), but only explained 1%, 0.5%, 0.2%, and 0.15% of the constrained variance, respectively.

When analyzing the samples collected at the second time point separately, 1.3% of the constrained variance of the microbiome of individual animal samples was statistically significantly explained by feedlot ID (*p* < 0.05). For pen-floor composite samples, the variables feedlot ID, total ADD exposure, and total MLS ADD exposure were statistically significant (*p* < 0.05) in the RDA of samples collected at the second time point, explaining 1.2%, 0.3%, and 0.3% of the constrained variance at the phylum level, respectively.

## Discussion

This unique study leveraged AMD use data from commercial beef feedlots in combination with state-of-the art target-enriched metagenomic sequencing and 16S rRNA gene sequencing to investigate critically important questions about factors affecting the promotion of AMR. Results suggested that AMD exposures in beef feedlot cattle do not strongly affect the fecal resistome or microbiome, compared to other factors measured in this study. Despite examination of 14 permutations of AMD exposure in this population that were derived from exceptionally detailed records of AMD exposures in individuals and in groups, these exposure variables were only significantly associated with explaining <1% of the variance in the composition of the resistome or microbiome. While the factor most strongly associated with resistome and microbiome composition was time in the feedlot, the majority of variability in the resistome composition remained unexplained. Indeed, the variance in the microbiome and resistome that can be explained by the exposure data in this study, or constrained variance, was much smaller than the variance that could not be modeled, unconstrained variance, so results should be interpreted with caution. Results of Procrustes analyses showed only moderate correlation between compositions of the microbiome and resistome features, and when stratifying on AMD exposure categories (Low vs. Medium vs. High), patterns in the Procrustes correlations were not consistent between individual animal samples and pen-floor composite samples. These findings suggest that the relationships between microbiome and resistome compositions were not predictably affected by AMD exposures in these populations.

This study was one of the largest conducted to date regarding the potential anthropogenic impact of AMD exposures typical of those used throughout the beef industry in North America as a promoter of AMR, as assessed using genomic sequencing. These findings add to the growing evidence suggesting that total AMD exposure is not associated with large shifts in the resistome or microbiome of cattle (Vikram et al., 2017; Doster et al., 2018; Rovira et al., 2019).

The higher prevalence of tetracycline, MLS, and aminoglycoside AMR determinants within the cattle resistome, and of Bacteroidota, Firmicutes, and Proteobacteria within the fecal microbiome of cattle is well documented (Ghosh and LaPara, 2007; Chen et al., 2008; Platt et al., 2008; Kyselková et al., 2015; Noyes et al., 2016b, 2017; Doster et al., 2018; Rovira et al., 2019). As such, their dominance within individual feedlot cattle and pen-floor composite samples collected in this study was unsurprising. Similarly, a greater variation in resistome and microbiome composition within individual animals upon feedlot arrival compared to pen-floor composite samples and later timepoints was expected, as cattle-associated microbial communities are influenced by the multitude of environmental pressures involved with transportation to a feedlot, initial processing, and diet changes typical of feedlot arrival (Noffsinger et al., 2015). Similar shifts in the resistome and microbiome diversity and composition of feces over time have been documented in previous studies of beef cattle during their transition to the feedlot environment (Checkley et al., 2008; Beukers et al., 2015; Noyes et al., 2016b; Doster et al., 2018). These changes are also consistent with the broader conclusion that the function and composition of host-associated microbial communities are significantly influenced by environmental factors (Shafquat et al., 2014). In particular, shifts in microbial community structure resulting from diet changes are well-documented (e.g., Singh et al., 2017; Ijaz et al., 2018; Youngblut et al., 2019), and it follows that shifts in resistome and microbiome composition observed in our study would occur as cattle and their associated microbial communities adapted to feedlot environmental pressures. Further, it stands to reason that the microbiome and resistome of pen-floor composite samples would contain less variation as the microbial community is influenced by contact with the soil microbiome over longer periods of times as pen-floors. Additionally, once cattle feces is exposed to the environment, the microbial community would be influenced by distinct factors such as UV light, pH, soil nutrients, moisture levels, and weather.

Despite the perception that AMD use in food animals is a contributor to AMR and treatment failure in humans, there is a paucity of data documenting that these AMD exposures in animals significantly change the resistome, and that resistance determinants are systematically transferred to humans through direct and indirect transmission routes (Robinson et al., 2016; Williams-Nguyen et al., 2016). Concerns regarding anthropogenic promoters of AMR have been especially strong regarding genes that can be found in medically important human pathogens, such as those of ESKAPE pathogens (Pendleton et al., 2013; Santajit and Indrawattana, 2016). The use of third generations cephalosporins, fluoroquinolones, and extended spectrum macrolides in cattle have been noted by some critics as being particularly concerning regarding risks to public health, and antimicrobial resistance determinants for these classes of AMDs, including those that are of high concern when found in medically important human pathogens (Supplementary material S8) were identified in these samples. However, antimicrobial use was not associated with the resistome composition or changes over the feeding period. Additionally, resistance determinants were identified for a number of important classes of AMDs that are not approved for use in cattle (e.g., aminoglycosides, carbapenems, streptogramins, lincosamides, linezolid, and pleuromutilins), and therefore antimicrobial use practices cannot directly explain the presence of these important AMR determinants.

We acknowledge that our study faces the same limitations of many high-throughput sequencing studies. Given the understated nature of the effects of AMD exposure, sequencing depth could have been inadequate to fully characterize the subtle dynamics occurring in low abundance features. However, use of target-enriched sequencing greatly enhanced depth of sequencing that can be efficiently achieved by typical shotgun sequencing, and numbers of reads associated with the resistome that were analyzed in this study are multiple logs greater than in previous studies (Noyes et al., 2016b, 2017; Weinroth et al., 2018; Rovira et al., 2019). The impact of this target-enrichment approach can be noted by the numbers of sequencing reads aligning to medically important genes in this study, which were much greater in number and allowed identification of broader range of important yet rare features than were identified in previous studies investigating the resistome in feedlot cattle (Noyes et al., 2016b; Weinroth et al., 2018; Rovira et al., 2019). Further, while this study serves as an example of how the use of metagenomics can produce significant and complimentary results from archived samples, it should be noted that samples were originally processed for aerobic culture and stored in Cary Blair media in a refrigerator prior to freezing. Therefore, our internal validity is sound, but comparisons to external studies using different methods of sample preprocessing should be made with caution.

A thorough search of the current literature yielded no other metagenomic studies investigating the impact of AMD exposure in beef feedlot cattle on the structure and function of microbial communities. This study substantiates evidence that prior AMD exposure may exert a subtle effect on the microbiome and resistome of feedlot cattle within an ecological context. These results further our understanding of how herd management decisions can influence the microbiome and the resistome, and provide data to help identify practices that maintain the critical balance between the benefits of AMD use and the risk of AMR emergence. As we learn to better manage AMR through livestock production practices, metagenomic analysis will be a critical tool for incorporating a holistic perspective into community-wide changes.

## Data availability statement

All sequence reads were made available through BioProject PRJNA755709 at the NCBI Sequence Read Archive. The code and instructions used in bioinformatic and statistical analyses can be found at this GitHub repository: https://github.com/Microbial-Ecology-Group/Antimicrobial-drug-use-effect-on-microbiome-and-resistome-of-beef-feedlots.

## Ethics statement

All cattle handling and sampling procedures were approved prior to the initiation of the study by the Animal Care Committee of the University of Calgary (Protocol Number M07031). Written informed consent was obtained from the owners for the participation of their animals in this study.

## Author contributions

PM, SG, TM, SH, and CB conceived the original research and organized and oversaw all aspects of its conduct, including collection of fecal samples and detailed antimicrobial drug use records from commercial beef feedlots. PM and KB conceived and oversaw all aspects of the follow-up research regarding metagenomic sequencing of fecal samples collected during the original research. SG and NN assisted in analyzing and preparing the antimicrobial drug exposure data for use in this study. JP and CA conducted laboratory procedures to extract DNA and prepare target-enriched shotgun sequencing libraries. ED analyzed the metagenomic sequencing data, conducted statistical analyses, and prepared initial drafts of the manuscript. ED, PM, KB, SG, and LP edited and prepared the final manuscript. All authors contributed to the article and approved the submitted version.

## Funding

This research was funded by grants from the USDA National Institute of Food and Agriculture (2015-68003-23048), Advancing Canadian Agriculture and Agri-Food (ACAAF) Program, Alberta Beef Producers (0007-038RDB), Canadian Cattlemen’s Association—Beef Cattle Research Council (BCRC 6.41), and by Texas A&M University.

## Conflict of interest

CB and SH were employed by Feedlot Health Management Services which was employed to manage the health of cattle studied for this research.

The remaining authors declare that the research was conducted in the absence of any commercial or financial relationships that could be construed as a potential conflict of interest.

## Publisher’s note

All claims expressed in this article are solely those of the authors and do not necessarily represent those of their affiliated organizations, or those of the publisher, the editors and the reviewers. Any product that may be evaluated in this article, or claim that may be made by its manufacturer, is not guaranteed or endorsed by the publisher.

## Supplementary material

The Supplementary material for this article can be found online at: https://www.frontiersin.org/articles/10.3389/fmicb.2022.970358/full#supplementary-material.

SUPPLEMENTARY FILE 1 Ranges used for making categorical variables.

SUPPLEMENTARY FILE 2 Resistome sequencing sample metadata.

SUPPLEMENTARY FILE 3 Microbiome sequencing sample metadata.

SUPPLEMENTARY FILE 4 Resistome sequencing count matrix.

SUPPLEMENTARY FILE 5 Count matrix of gene accessions requiring SNP confirmation.

SUPPLEMENTARY FILE 6 Differential abundance testing results for individual animal samples at the AMR class level, by sampling time.

SUPPLEMENTARY FILE 7 Differential abundance testing results for composite pen-floor samples at the AMR mechanism level, by sampling time.

SUPPLEMENTARY FILE 8 Count matrix of highly important AMR genes.

SUPPLEMENTARY FILE 9 Microbiome sequencing count matrix.

SUPPLEMENTARY FILE 10 Differential abundance testing results for individual animal samples at the microbiome phylum level, by sampling time.

SUPPLEMENTARY FILE 11 Differential abundance testing results for composite pen-floor samples at the microbiome phylum level, by sampling time.

SUPPLEMENTARY FIGURE 1 Sample selection diagram for **(A)** individual animal fecal samples and **(B)** composite pen-floor samples.

SUPPLEMENTARY FIGURE 2 Resistome rarefaction for all individual animal samples at the gene accession level.

SUPPLEMENTARY FIGURE 3 Microbiome rarefaction for all individual animal samples at the ASV level.

SUPPLEMENTARY FIGURE 4 Resistome rarefaction for all composite pen-floor samples at the gene accession level.

SUPPLEMENTARY FIGURE 5 Microbiome rarefaction for all composite pen-floor samples at the ASV level.

SUPPLEMENTARY FIGURE 6 Heatmap of counts to highly important AMR genes identified in this study.

## Footnotes

^1^ https://megares.meglab.org

## Abbreviations

ADD: Animal defined daily dose
AMD: Antimicrobial drug
AMR: Antimicrobial resistance
ASV: Amplicon sequence variants
CDC: Centers for Disease Control
CSS: Cumulative sum scaling
DOF: Days on feed
MDR: Multi-drug resistance
MFS: Major facilitator superfamily
MLS: Macrolide-lincosamide-streptogramin
NGS: Next-generation sequencing
RDA: Redundancy analysis
WHO: World Health Organization
