# Supplementary figures and images for: Evaluating the effects of antimicrobial drug use on the ecology of antimicrobial resistance and microbial community structure in beef feedlot cattle
Source: Front Microbiol. 2022 Dec 13;13:970358. doi: 10.3389/fmicb.2022.970358 (PMC9792868; doi:10.3389/fmicb.2022.970358)

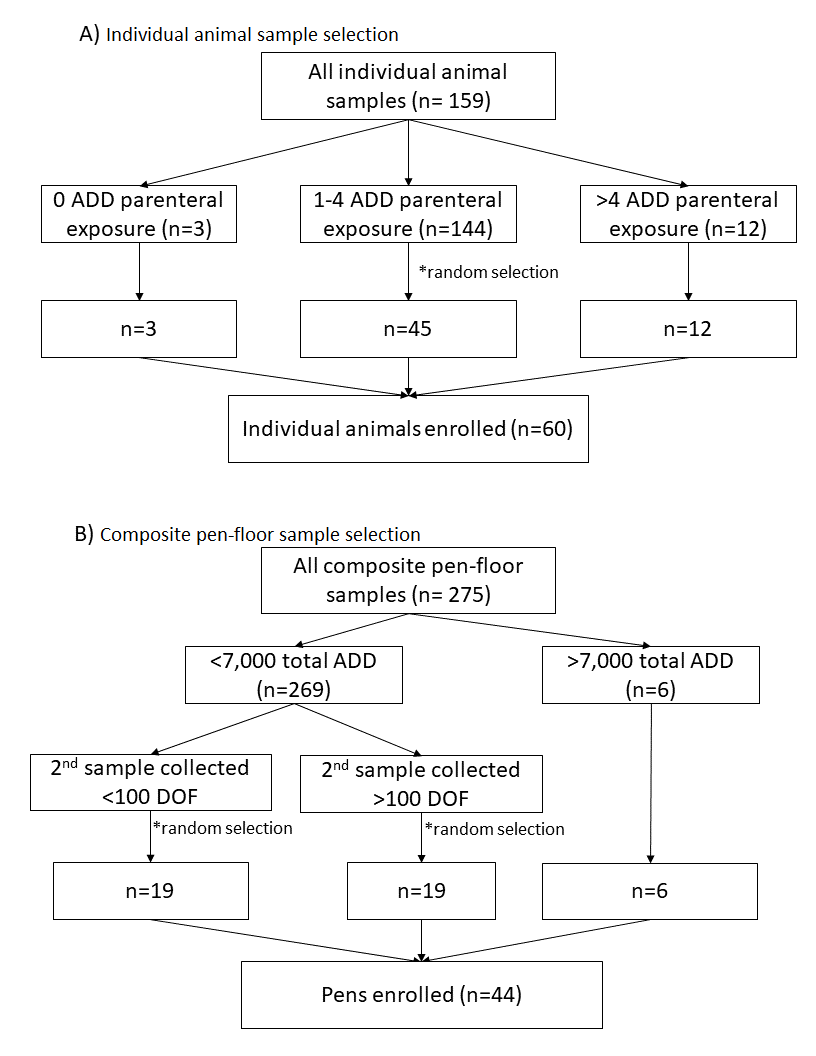

Supplement: SUPPLEMENTARY FIGURE 1 — Sample selection diagram for (A) individual animal fecal samples and (B) composite pen-floor samples. [file Image_1.png]

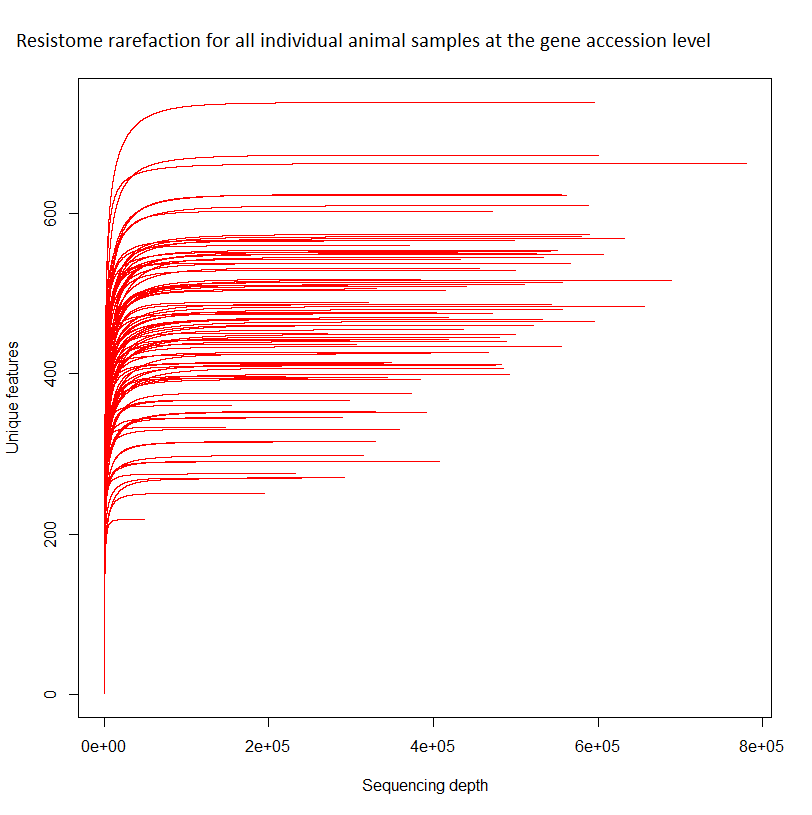

Supplement: SUPPLEMENTARY FIGURE 2 — Resistome rarefaction for all individual animal samples at the gene accession level. [file Image_2.png]

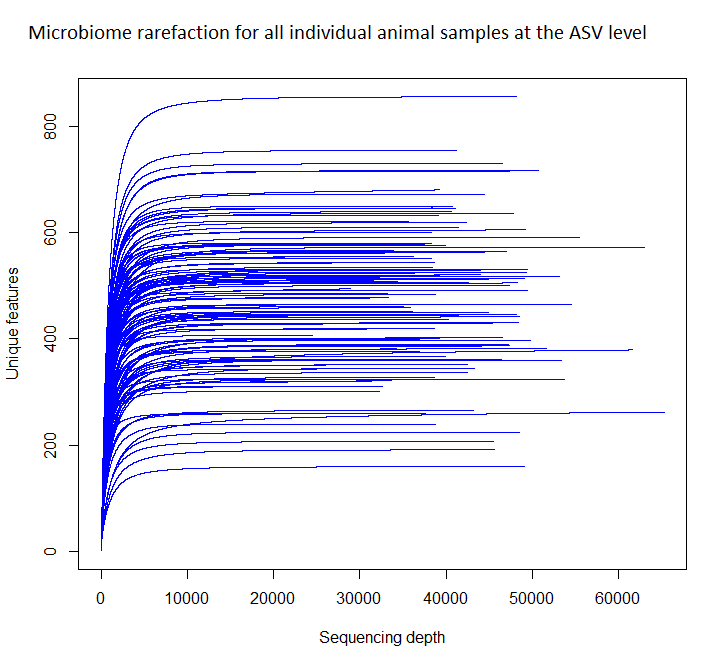

Supplement: SUPPLEMENTARY FIGURE 3 — Microbiome rarefaction for all individual animal samples at the ASV level. [file Image_3.png]

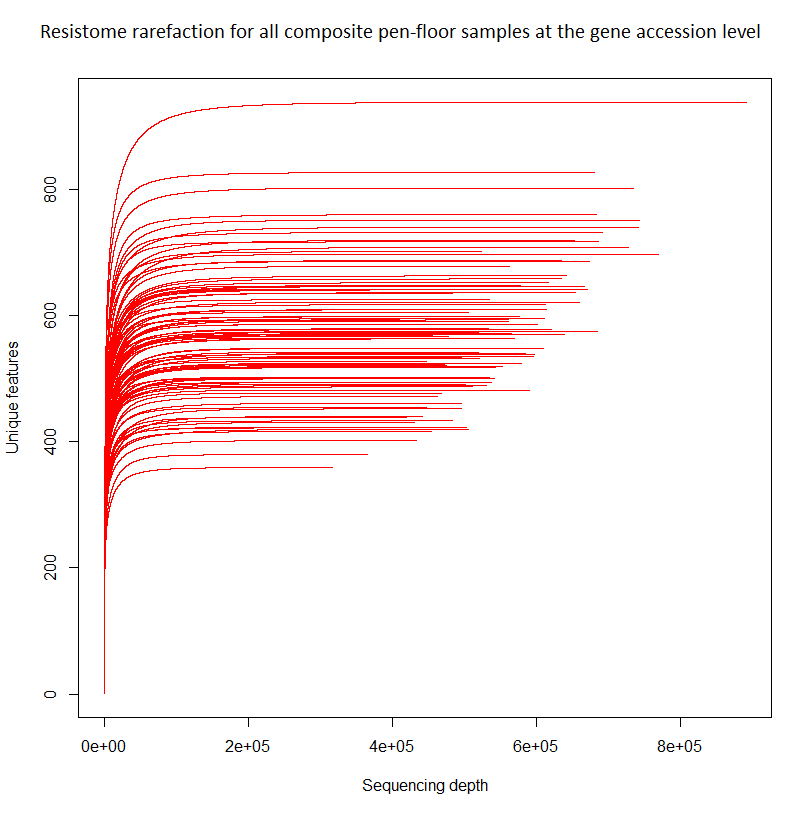

Supplement: SUPPLEMENTARY FIGURE 4 — Resistome rarefaction for all composite pen-floor samples at the gene accession level. [file Image_4.png]

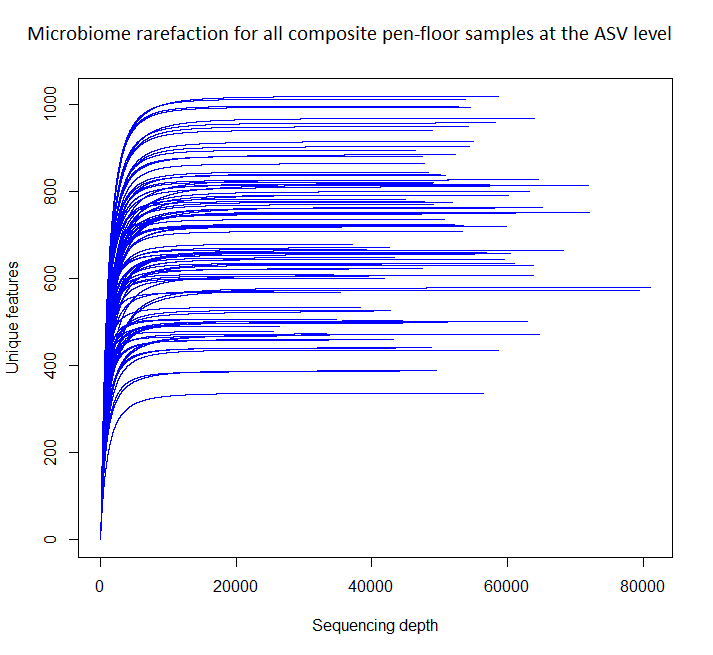

Supplement: SUPPLEMENTARY FIGURE 5 — Microbiome rarefaction for all composite pen-floor samples at the ASV level. [file Image_5.png]

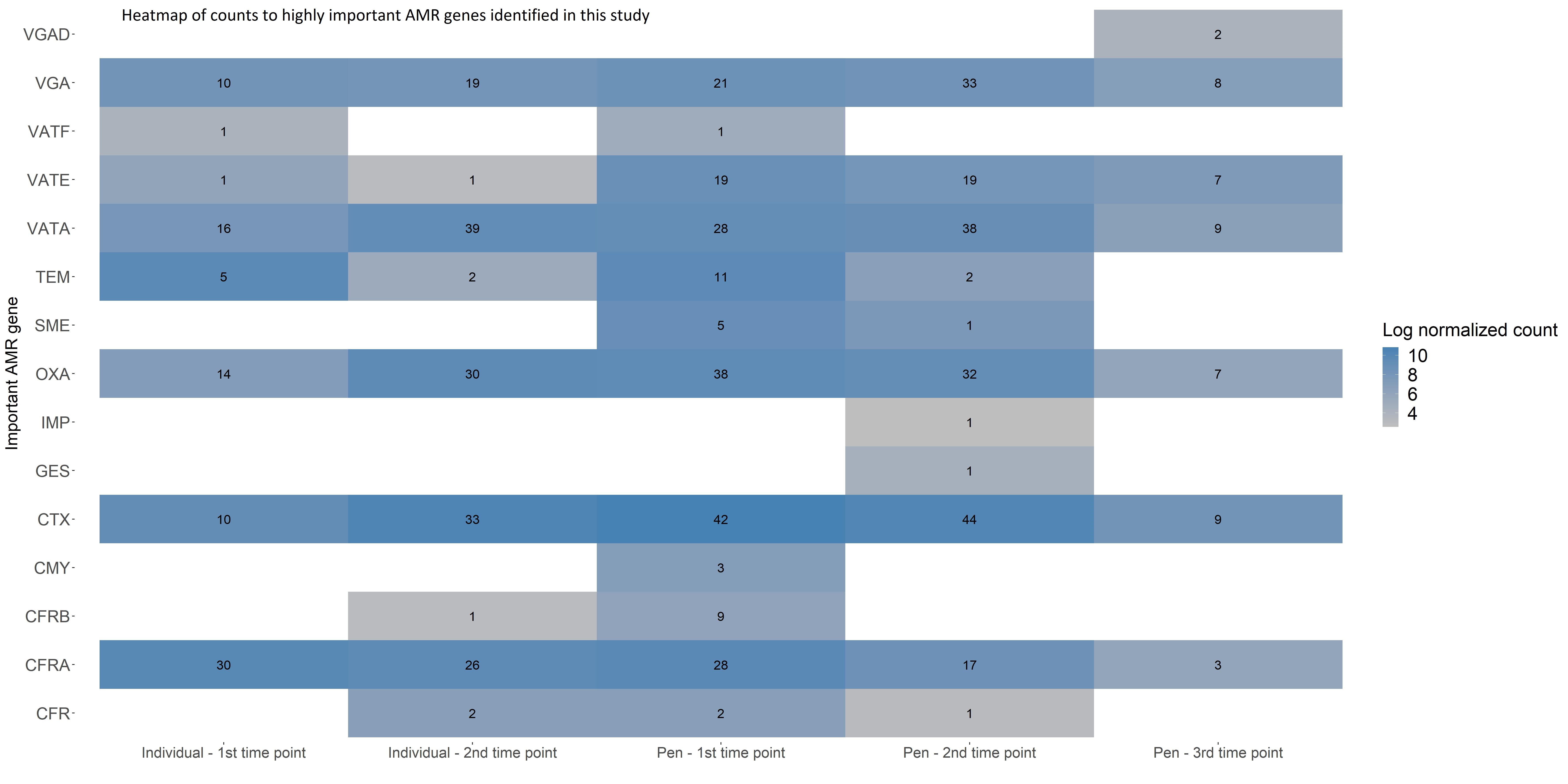

Supplement: SUPPLEMENTARY FIGURE 6 — Heatmap of counts to highly important AMR genes identified in this study. [file Image_6.jpeg]
